# Identification of key gene networks controlling polysaccharide accumulation in different tissues of *Polygonatum cyrtonema* Hua by integrating metabolic phenotypes and gene expression profiles

**DOI:** 10.3389/fpls.2022.1012231

**Published:** 2022-09-29

**Authors:** Longsheng Chen, Shuwen Xu, Yujun Liu, Yanhong Zu, Fuyuan Zhang, Liji Du, Jun Chen, Lei Li, Kai Wang, Yating Wang, Shijin Chen, Ziping Chen, Xianfeng Du

**Affiliations:** ^1^Anhui Engineering Laboratory for Agro-Products Processing, School of Tea & Food Science and Technology, Anhui Agricultural University, Hefei, China; ^2^Anhui Promotion Center for Technology Achievements Transfer, Anhui Academy of Science and Technology, Hefei, China; ^3^Jinzhai Senfeng Agricultural Technology Development Co., Ltd., Lu’an, China

**Keywords:** polysaccharides, sucrose metabolism and transport, transcription factors, gene expression, *Polygonatum cyrtonema* Hua

## Abstract

Plant polysaccharides, a type of important bioactive compound, are involved in multiple plant defense mechanisms, and in particular polysaccharide-alleviated abiotic stress has been well studied. *Polygonatum cyrtonema* Hua (*P. cyrtonema* Hua) is a medicinal and edible perennial plant that is used in traditional Chinese medicine and is rich in polysaccharides. Previous studies suggested that sucrose might act as a precursor for polysaccharide biosynthesis. However, the role of sucrose metabolism and transport in mediating polysaccharide biosynthesis remains largely unknown in *P. cyrtonema* Hua. In this study, we investigated the contents of polysaccharides, sucrose, glucose, and fructose in the rhizome, stem, leaf, and flower tissues of *P. cyrtonema* Hua, and systemically identified the genes associated with the sucrose metabolism and transport and polysaccharide biosynthesis pathways. Our results showed that polysaccharides were mainly accumulated in rhizomes, leaves, and flowers. Besides, there was a positive correlation between sucrose and polysaccharide content, and a negative correlation between glucose and polysaccharide content in rhizome, stem, leaf, and flower tissues. Then, the transcriptomic analyses of different tissues were performed, and differentially expressed genes related to sucrose metabolism and transport, polysaccharide biosynthesis, and transcription factors were identified. The analyses of the gene expression patterns provided novel regulatory networks for the molecular basis of high accumulation of polysaccharides, especially in the rhizome tissue. Furthermore, our findings explored that polysaccharide accumulation was highly correlated with the expression levels of *SUS*, *INV*, *SWEET*, and *PLST*, which are mediated by *bHLH*, *bZIP*, *ERF*, *ARF*, *C2H2*, and other genes in different tissues of *P. cyrtonema* Hua. Herein, this study contributes to a comprehensive understanding of the transcriptional regulation of polysaccharide accumulation and provides information regarding valuable genes involved in the tolerance to abiotic stresses in *P. cyrtonema* Hua.

## Introduction

**FIGURE 1:**
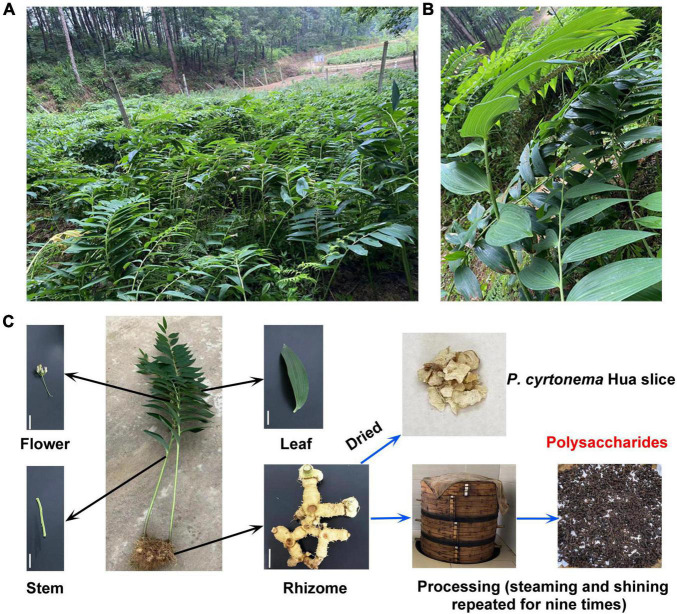
Growth environment, phenotype, and processing of *Polygonatum cyrtonema* (*P. cyrtonema*) Hua. **(A)** *P. cyrtonema* Hua is often grown on the mountainside by underwood planting. **(B,C)** The phenotype of the whole and different parts of *P. cyrtonema* Hua. Bar = 5 cm. To date, steaming and drying are the most used methods in the processing of rhizome of *P. cyrtonema* Hua. Polysaccharide (PCP) is one of the most crucial compounds of processed product and contributes to flavor and has health benefits.

*Polygonati rhizoma*, commonly known as Huangjing, is a vital traditional and classic Chinese herb and is widely distributed in the world (Luo et al., 2016). There are various species, including *Polygonatum cyrtonema* Hua, *Polygonatum kingianum* Collett and Hemsley, and *Polygonatum sibiricum* Red et al in China (Chinese Pharmacopoeia Commission, 2020). Among these, *Polygonatum cyrtonema* Hua (*P. cyrtonema* Hua) is a key constituent of herbal medicines in Anhui Province. Indeed, it usually grows on forested mountains, bushes, and shade hillsides at an altitude of 500–1,200 m above sea level (Figures 1A,B). *P. cyrtonema* Hua often blooms from late April to early June, and the perianth shows the yellow-green color (Figures 1B,C). Its fruit ripens around the middle of August. Moreover, the rhizomes are harvested in late October and are used to manufacture the Huangjing slice, nine cycles of steam-sun drying processing products, functional beverages, fruit wine, and well-received sweetmeats (Figure 1C; Chinese Pharmacopoeia Commission, 2020; Li et al., 2021; Wu et al., 2022). They have a high abundance of health benefit-promoting metabolites and are among the widely consumed medicines and functional foods (Cheng et al., 2021; Li et al., 2021; Hu et al., 2022; Zhu et al., 2022).

**FIGURE 2:**
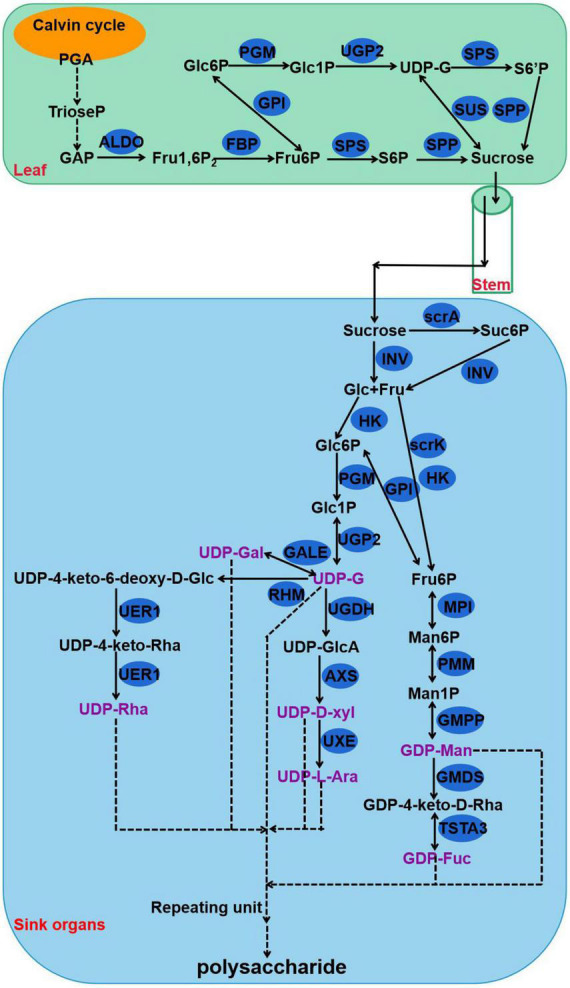
The proposed pathway for the biosynthesis of polysaccharides in *Polygonatum cyrtonema*. 3-phosphoglycerate, PGA; Triose phosphate, TrioseP; Glyceraldehyde-3-phosphate, GAP; fructose-1,6-diphosphatase, Fru1,6P; Fructose 6-phosophate, Fru-6P; Sucrose 6-phosophate, S6P; Glucose, Glc; Fructose, Fru; Glucose 6-phosophate, Glc-6P; Glucose 1-phosophate, Glc1P; Sucrose 6′-phosophate, S6′P; Uridine diphosphate glucose, UDP-G; Mannose-6-phosphate, Man6P; Mannose-1-phosphate, Man1P; Guanosine diphosphate mannose, GDP-Man; UDP-4keto-D-rhamnose, GDP-4keto-D-Rha; Guanosine diphosphate fucose, GDP-Fuc; UDP-glucuronic acid, UDP-GlcA; UDP-D-xylose, UDP-D-xyl; UDP-L-arabinose, UDP-L-Ara; UDP-galactose, UDP-Gal; UDP-4-keto-6-deoxy-D-Glucose, UDP-4-keto-6-deoxy-D-Glc; UDP-4-keto-rhamnose, UDP-4-keto-Rha; UDP-rhamnose, UDP-Rha; fructose-bisphosphate aldolase, ALDO; fructose-1,6-bisphosphatase, FBP; glucose-6-phosphate isomerase, GPI; phosphoglucomutase, PGM; UTP-glucose-1-phosphate uridylyltransferase, UGP2; sucrose synthase, SUS; sucrose PTS system EIIBCA or EIIBC component, scrA; sucrose-phosphate synthase, SPS; sucrose-6-phosphatase, SPP; invertase, INV; sucrose synthase, SUS; hexokinase, HK; fructokinase, scrK; mannose-6-phosphate isomerase, MPI; phosphomannomutase, PMM; mannose-1-phosphate guanylyltransferase, GMPP; GDPmannose 4,6-dehydratase, GMDS; GDP-L-fucose synthase, TSTA3; UDP-glucose 4-epimerase, GALE; UDP-glucose 6-dehydrogenase, UGDH; UDP-apiose/xylose synthase, AXS; UDP-arabinose 4-epimerase, UXE; UDP-glucose 4,6-dehydratase, RHM; 3,5-epimerase/4-reductase, UER1.

Polysaccharides of *P. cyrtonema* Hua are important bioactive compounds (Li et al., 2021). Numerous pharmaceutical results revealed that polysaccharides of rhizomes have a large set of functions, such as anti-oxidation, anti-herpes, antibacterial, anti-cancer, lowering of blood sugar levels, and lowering of blood lipids, as well as the lung-protective and anti-viral effects (Liu et al., 2004; Li et al., 2018a,b, 2020, 2021; Xie et al., 2020; Gan et al., 2022). For example, polysaccharides that are sequentially extracted from *P. cyrtonema* Hua exhibited anti-cancerous function against cervical cancer Hela cells *via* regulating the cell cycle and the genes related to different apoptosis pathways which are involved in Hela cell cycle arrest and apoptosis (Li et al., 2020). Previous studies also indicated that polysaccharides can act as compatible solutes and enhance abiotic stress tolerance, such as drought and salt stress in plants (Yu et al., 2017, 2021). In recent years, intermediate metabolites and genes related to polysaccharide biosynthesis have been explored in various plants, including *Arabidopsis*, wheat, rice, *Cyclocarya paliurus*, *Cynomorium songaricum*, *Dendrobium officinale*, and *Polygonatum odoratum* (Northcote and Pickett-Heaps, 1966; Meng et al., 2009; Oh et al., 2013; Zhang et al., 2016; He et al., 2017; Zhang et al., 2020; Lin et al., 2021; Wang et al., 2021a). Studies have discovered that polysaccharides are biosynthesized *via* continuous enzymatic reactions from sucrose, requiring invertase (INV), hexokinase (HK), fructokinase (scrK), mannose-6-phosphate isomerase (MPI), phosphomannomutase (PMM), mannose-1-phosphate guanylyltransferase (GMPP), GDP-mannose 4,6-dehydratase (GMDS), GDP-L-fucose synthase (TSTA3), UDP-glucose 4-epimerase (GALE), UDP-glucose 6-dehydrogenase (UGDH), UDP-apiose/xylose synthase (AXS), UDP-arabinose 4-epimerase (UXE), UDP-glucose 4,6-dehydratase (RHM), 3,5-epimerase/4-reductase (UER1), and glycosyltransferase (GT) (Figure 2; Zhang et al., 2020). First, sucrose is converted to glucose (Glc) and fructose (Fru) by INV. Then, HK and scrK convert Glc and Fru to glucose-6-phosphate (Glc-6P) and fructose-6-phosphate (Fru-6P), respectively. Later, they are used to synthesize UDP-glucose (UDP-Glc) and guanosine di phosphomannose (GDP-Man). A series of glycosyltransferase (GT) reactions result in the production of polysaccharides.

Recently, some molecular studies on the genes related to polysaccharide biosynthesis of *P. cyrtonema* Hua have been published (Wang et al., 2019; Li et al., 2022). For example, Wang et al. (2019) speculated the multiple genes encoding important enzymes, such as UDP glycosyltransferases (UGTs), and the transcription factors through comprehensive analyses of the transcriptome in leaf, root, and rhizome tissues. Besides, a positively correlated link between polysaccharide accumulation and the *MPI*, *TSTA3*, *AXS*, and *UGDH* transcript levels was also suggested *via* the transcriptome sequencing for rhizomes at different ages (Li et al., 2022).

Plant leaves capture light energy and convert this to carbohydrates, such as 3-phosphoglycerate (PGA) and glyceraldehyde-3-phosphate (GAP), during the process of photosynthesis (Osorio et al., 2014). Then, sucrose is synthesized by sucrose synthase (SUS), sucrose-phosphate synthase (SPS), and sucrose-6-phosphatase (SPP) in the chloroplast during the daytime (Figure 2; Osorio et al., 2014). Other enzymes, such as fructose-bisphosphate aldolase (ALDO), fructose-1,6-bisphosphatase (FBP), glucose-6-phosphate isomerase (GPI), phosphoglucomutase (PGM), and UTP-glucose-1-phosphate uridylyltransferase (UGP2), are also involved in the pathway of sucrose synthesis (Figure 2). Plants have evolved a complex system to balance the distribution and allocation of carbon (C) (Raines and Paul, 2006; Kircher and Schopfer, 2012). For example, sucrose metabolism is vital for both the allocation of crucial carbon resources and the initiation of hexose-based sugar signals (Koch, 2004). As a pivotal energy substance, sucrose is transported through the vasculature, type, and sink tissues by *SUTs/SUCs* (sucrose transporters or sucrose carriers) and/or *SWEETs* (Sugars Will Eventually be Exported Transporters) and serves as a metabolite for plant growth and development (Feng et al., 2019; Mathan et al., 2021a). These sugar metabolism and transport pathways are mainly controlled by transcription factors, such as *bZIP*, *WRKY*, *bHLH*, *AP2/ERF*, *NAC*, and *MYB* (Sun et al., 2003; Zhu et al., 2011; Phukan et al., 2018; Yang et al., 2021; Wang et al., 2021b; Zhang et al., 2022). Besides, this transporter-regulated sucrose accumulation played a specific role in plant abiotic stress tolerance and adaptation (Chandran, 2015; Eggert et al., 2016). Interestingly, in the rhizome of *P. cyrtonema* Hua, sucrose might act as a precursor for polysaccharide biosynthesis (Wang et al., 2019; Li et al., 2022). However, whether or not, or how polysaccharide accumulation in rhizomes is modulated by sucrose synthesis and transported in leaf and/or stem remains unknown in *P. cyrtonema* Hua.

In this study, we investigated the polysaccharides, sucrose, glucose, and fructose content in different parts (rhizome, stem, leaf, and flower) of *P. cyrtonema* Hua. By the whole-transcriptome analysis, we performed a systematic analysis of polysaccharide biosynthetic genes and estimated the transcript levels of these genes in rhizome, stem, leaf, and flower tissues. Furthermore, several sugar transporters, such as *SWEETs*, *ERDs*, and *SUTs*, were involved in sucrose transport from leaves to stem, rhizome, and flower tissues, thereby coordinately the regulation of the polysaccharide biosynthesis, especially in the rhizome. Finally, we filtrated some key transcription factors, such as *bZIP*, *bHLH*, and *ERF*, which might regulate the above genes related to sucrose metabolism and transport. Briefly, our findings would provide the potential key regulatory points of polysaccharide accumulation in *P. cyrtonema* Hua.

## Materials and methods

### Plant materials and growth conditions

Plants of *P. cyrtonema* Hua were collected on May 20 from the *P. cyrtonema* Hua germplasm conservation and breeding base at Jinzhai County in Anhui Province, China. They were identified by Associate Professor Yujun Liu (Anhui Promotion Center for Technology Achievements Transfer, Anhui Academy of Science and Technology). Plants were cleaned and dried on filter paper, and then imaged using a Canon IXUS 130 camera. Meanwhile, rhizomes, leaves, stems, and flower parts were sampled, and immediately frozen in liquid nitrogen and stored at −80°C.

### Measurement of the polysaccharide, glucose, fructose, and sucrose content

Rhizomes, leaves, stems, and flower parts of *P. cyrtonema* Hua were oven-dried at 70°C to constant weight. Then, the dried samples were ground into powder using the grinding mill and kept in a glassware dryer, and further used for the determination of polysaccharide, glucose, fructose, and sucrose content.

Total polysaccharides were extracted and detected from different dried tissues of *P. cyrtonema* Hua by the anthrone-sulfuric acid method in the Chinese Pharmacopoeia Commission (2020). Briefly, 0.2 g of samples was weighed into a round-bottom flask with 120 mL of 80% ethanol, and boiled for 1 h to remove the small organic compounds and pigments. Afterward, 120 mL of distilled water was added and extracted at 80°C for another 1 h. The supernatant was transferred and diluted in a 250 mL volumetric flask. Polysaccharide content was determined spectrophotometrically by measuring the absorbance at 582 nm with 1 mL of extract, 1 mL of distilled water, and 8 mL of 0.2% anthrone-sulfuric acid solution. The concentration was calculated according to the standard curve of glucose.

Sucrose in different tissues of *P. cyrtonema* Hua was extracted and determined as previously described (Feng et al., 2019). Samples were homogenized with 1 mL of extracting solution, heated to 80°C in a water bath for 10 min, and then centrifuged (10,000 × *g*; 25°C) for 15 min. About 5 mg of activated carbon was added to the supernatant for decoloring at 80°C for 30 min. After the solution was cooled to room temperature, 60 μL of 0.1 M NaOH solution was added to 100 μL of the supernatant in a 100°C water bath for 5 min. Then, 700 μL of 10 M HCl and 200 μL of 0.1% resorcinol were added to a 100°C water bath for 10 min. After cooling, the sucrose content was determined and calculated by measuring the absorbance at 480 nm.

Glucose in different tissues of *P. cyrtonema* Hua was extracted and determined using the Glucose Assay Kit (Solarbio Life Sciences, Beijing, China) according to the manufacturer’s instructions. Samples were homogenized with 1 mL of H_2_O and heated to 80°C in a boiling water bath for 10 min, and then centrifuged (10,000 × *g*; 25°C) for another 10 min. Glucose concentration was determined by a glucose oxidase enzyme system and calculated by measuring the absorbance at 505 nm.

Fructose in different tissues of *P. cyrtonema* Hua was extracted and determined using the Fructose Assay Kit (Solarbio Life Sciences, Beijing, China) according to the manufacturer’s instructions. Samples were homogenized with 1 mL of extracting solution, heated to 80°C in a water bath for 10 min, and then centrifuged (10,000 × *g*; 25°C) for 10 min. About 5 mg of activated carbon was added to the supernatant for decoloring at 80°C for 30 min. After the solution cooled to room temperature, 1.4 mL of 10 M HCl and 400 μL of 0.1% resorcinol were added to 200 μL of supernatant in a 100°C water bath for 10 min. After cooling, the fructose content was determined and calculated by measuring the absorbance at 480 nm.

### Total RNA extraction, cDNA library construction, and sequencing

Total RNA of different tissues (rhizome, stem, leaf, and flower) of P. cyrtonema Hua was extracted using the Trizol reagent (Invitrogen, Carlsbad, CA, USA) according to the manufacturer’s instructions. RNA concentration and quality were checked using the NanoDrop 2000 (Thermo Fisher Scientific, Inc., Waltham, MA, USA). Sequencing libraries were generated using the TruSeq RNA Sample Preparation Kit (Illumina Inc., San Diego, CA, USA) by using three biological replicates according to the manufacturer’s instructions. Then, the libraries were sequenced using the next-generation sequencing (NGS) and Illumina HiSeq. Enrichment of mRNA, fragment interruption, addition of adapters, size selection, CR amplification, and RNA-Seq were performed by staff at Personal Biotechnology Co., Ltd., Shanghai, China.

### Differential gene expression analysis

After the data filtration, the unigene set of sequences was constructed by the clean reads with high-quality samples using Trinity v2.10.0. The fragments per kilobase million (FPKM) of transcript sequence presented the expression level of each gene in each library. To determine the transcriptional changes among different parts of *P. cyrtonema* Hua, differentially expressed genes (DEGs) were identified by comparing the expression levels in rhizome vs. stem, rhizome vs. leaf, and rhizome vs. flower, respectively. Transcripts with | log_2_FoldChange| > 1 and *P*-value < 0.05 were considered as the thresholds for significant differences, which was performed by DEGseq 2.

### Kyoto encyclopedia of genes and genome and gene ontology enrichment analysis

All unigenes were annotated against the Kyoto Encyclopedia of Genes and Genome (KEGG) and Gene ontology (GO). KEGG annotation^1^ was performed to identify the metabolic pathways of genes. GO classifications^2^ were obtained according to molecular function, biological process, and cellular component. Over-represented KEGG and GO terms were detected *via* Fisher’s exact test, and multi-test adjustment was made using the Yekutieli (FDR under dependency) method with a cutoff of FDR < 0.05.

### Statistical analysis

Statistical analysis was performed using SPSS 18.0 software. Values were mean values and SD of three independent experiments with three replicates for each. For statistical analysis, data were analyzed by one-way analysis of variance (ANOVA) followed by Duncan’s multiple range test. *P*-values < 0.05 were considered statistically significant. The correlation coefficient between polysaccharide, sucrose, glucose, and fructose content and gene expression levels was analyzed *via* Pearson’s correlation coefficient and Student’s *t*-test (*P < 0.05, **P < 0.01, ***P < 0.001).

## Results

### Sugar composition in rhizome, stem, leaf, and flower tissues of *Polygonatum cyrtonema* Hua

**FIGURE 3:**
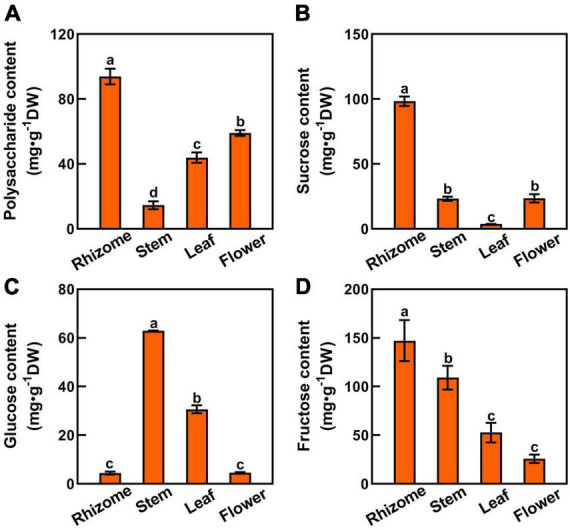
Sugar composition in different parts of *P. cyrtonema*. **(A–D)** Polysaccharide, sucrose, glucose, and fuctose content of rhizome, stem, leaf, and flower tissues of *P. cyrtonema*. Data represent mean ± SD (*n* = 3). Bars with different letters denote significant differences at *P* < 0.05 according to Duncan’s multiple comparison test.

Since sucrose is the major transportable form of photosynthate from source leaves to sink organs, we tested the polysaccharides, sucrose, glucose, and fructose contents in rhizome, stem, leaf, and flower tissues to unravel the mechanism underlying the polysaccharide biosynthesis in *P. cyrtonema* Hua (Figures 1, 3). Among them, the polysaccharide content of rhizome was the highest (93.87 mg⋅g^–1^DW), followed by flower (59.03 mg⋅g^–1^DW), and lowest in stem (14.51 mg⋅g^–1^DW) (Figure 3A). We also found that sucrose and fructose levels were higher in the rhizome than in the stem, leaf, and flower (Figures 3B,D). In contrast, the glucose content of the stem was the highest, followed by the leaf (Figure 3C). It was lowest in both rhizome and flower of *P. cyrtonema* Hua (Figure 3C). The above results clearly suggested that the polysaccharides are synthesized in rhizome, stem, leaf, and flower tissues. Meanwhile, the biosynthesis and transport of sucrose, glucose, and fructose in the leaf and stem significantly impacted the polysaccharide accumulation in the rhizome and flower of *P. cyrtonema* Hua.

### Transcriptional analysis showed the overview of gene expression profiles among the different tissues

To elucidate the molecular mechanisms of polysaccharide biosynthesis, a genome-wide transcriptional analysis was performed in the rhizome, stem, leaf, and flower tissues of *P. cyrtonema* Hua. For transcriptomic analysis, total RNA was extracted and used to prepare cDNA libraries by using three biological replicates. After processing, the raw sequence reads (between 42,336,126 and 52,068,538 clean reads in total) were aligned, and the Q20% (the percentage of bases with a Phred value >20) value for the clean reads was >97% and the Q30% (the percentage of bases with a Phred value >30) value of the clean reads was >92% and were found to map (Supplementary Table 1). A total of 182,700 unigenes were assembled. With regard to the unigenes, the total length and mean length of the unigenes were 157,174,631 and 860.29 bp, respectively, and their GC% was 44.78% (Supplementary Table 2). These results implied that high-quality sequencing data were obtained for the following analyses.

**FIGURE 4:**
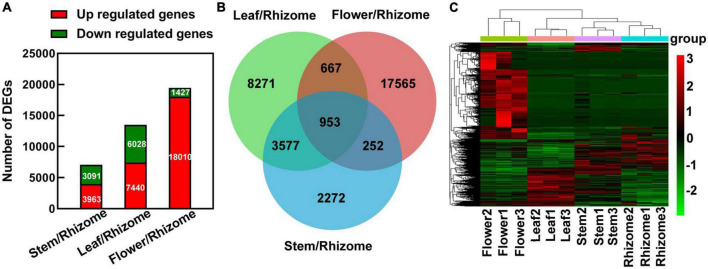
An overview of differentially expressed genes (DEGs) responsive to different parts of *Polygonatum cyrtonema*. **(A)** The number of DEGs was examined in the comparison between rhizome and stem, leaf, or flower. **(B)** Venn diagram showing the distribution of DEGs in the comparison between rhizome and stem, leaf, or flower. **(C)** Hierarchical clustering represents relative expression levels of DEGs in comparison between rhizome and stem, leaf, or flower. Multiple of differential expression [foldChange (Case/Control)]: foldChange (stem/rhizome), foldChange (leaf/rhizome), and foldChange (flower/rhizome). log_2_FoldChange > 1, *P*-value < 0.05. Red indicates upregulated genes, and green indicates downregulated genes.

In order to construct a detailed regulatory network for polysaccharide biosynthesis in different tissues, we compared differentially expressed genes (DEGs) between stem and rhizome, leaf and rhizome, and flower and rhizome, respectively. The DEGs were filtered by expression levels | log 2 (fold-change)| > 1 and FDR < 0.05 in each pairwise comparison, thus revealing 7,054 (3,963 up- and 3,091 downregulated) DEGs in stem/rhizome, 13,468 (7,440 up- and 6,028 downregulated) DEGs in leaf/rhizome, and 19,437 (18,010 up- and 1,427 downregulated) DEGs in flower/rhizome (Figure 4A). Then, we constructed a Venn diagram to investigate the numbers of co-expressed and uniquely expressed DEGs in each pairwise comparison. A total of 953 co-expressed DEGs were obtained among these (Figure 4B). Additionally, hierarchical clustering analysis showed that strong changes in DEG expression levels were observed in the different tissues (Figure 4C).

### Functional annotation and expression overview of unigenes

The above-assembled unigenes were searched and annotated in NR, GO, KEGG, PFAM, eggNOG, and Swiss prot databases using the BLAST algorithm. Our results showed that 49.47, 21.77, 20.66, 30.53, 40.79, and 37.27% unigenes were annotated as significant hits in NR, GO, KEGG, PFAM, eggNOG, and Swiss prot databases, respectively (Supplementary Table 3).

**FIGURE 5:**
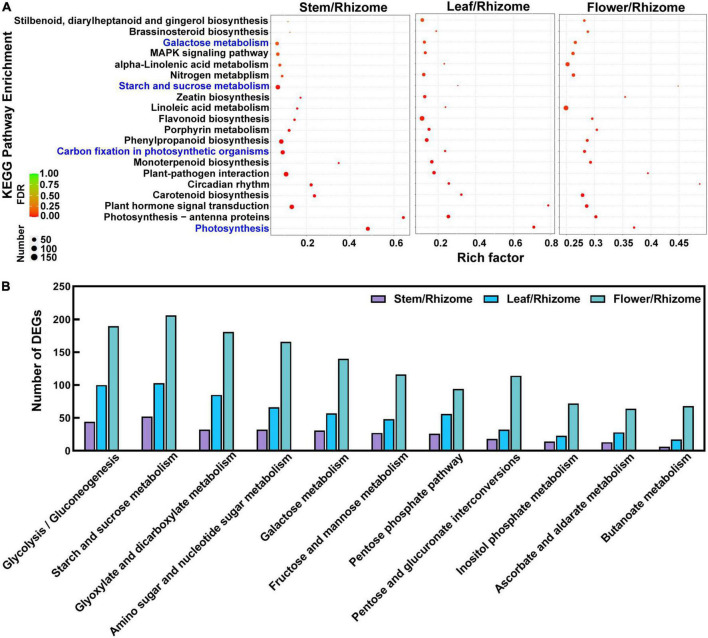
Functional enrichment analysis of differentially expressed genes (DEGs) by Kyoto Encyclopedia of Genes and Genomes (KEGG) pathway-based enrichment analysis. **(A)** The top 20 pathway classifications with the smallest FDR value were selected by KEGG pathway-based enrichment analysis due to the DEGs between rhizome and stem, leaf, or flower group. **(B)** The 11 pathway classifications for carbohydrate metabolism. Rich factor indicates the matched gene ratio, the circle size is proportional to the target gene number, and 0 < FDR (false discovery rate) <1.

**FIGURE 6:**
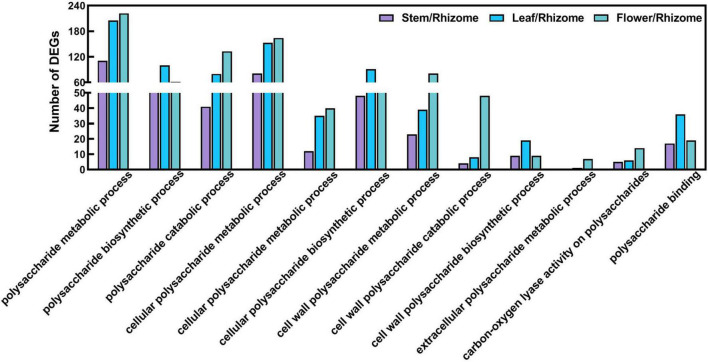
Functional enrichment analysis of differentially expressed genes (DEGs) by Gene Ontology (GO) pathway-based enrichment analysis. The 12 pathway classifications for the polysaccharide metabolism were selected by GO pathway-based enrichment analysis due to the DEGs between rhizome and stem, leaf, or flower group.

To understand the biological processes and pathways involved in polysaccharide biosynthesis, Kyoto Encyclopedia of Genes and Genomes (KEGG) and Gene Ontology (GO) pathway-based enrichment analyses were performed (Figures 5, 6). The results showed that the top 20 pathway classifications with the smallest FDR value were selected by KEGG pathway-based enrichment analysis due to the DEGs between the rhizome and stem, leaf, or flower group (Figure 5A). Among these, genes related to photosynthesis, carbon fixation in photosynthetic organisms, galactose metabolism, and starch and sucrose metabolism processes were significantly enriched. Then, we screened 15 pathway classifications from the DEGs related to carbohydrate metabolism, which are most likely associated with polysaccharide biosynthesis, such as starch and sucrose metabolism, fructose and mannose metabolism, glycolysis/gluconeogenesis, and pentose and glucuronate interconversions (Figure 5B and Supplementary Table 4). For example, there were 52, 103, and 206 DEGs involved in starch and sucrose metabolism among stem/rhizome, leaf/rhizome, and flower/rhizome, respectively. Moreover, GO pathway analysis revealed that there were numerous DEGs in the polysaccharide metabolic process, including cellular/cell wall polysaccharide biosynthetic and catabolic processes, directly related to polysaccharide biosynthesis in *P. cyrtonema* Hua (Figure 6 and Supplementary Table 5).

### Identification of genes involved in polysaccharide biosynthesis

**FIGURE 7:**
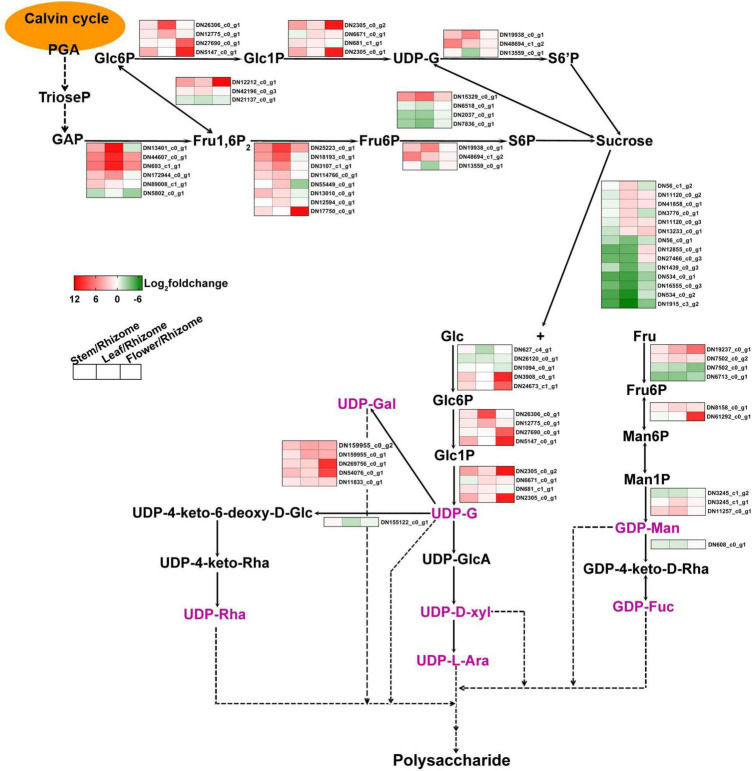
Heat map illustrating major expression profiles of differentially expressed genes (DEGs) involved in polysaccharide biosynthesis. Red indicates high expression, white indicates intermediate expression, and green indicates low expression.

To further comprehend the polysaccharide biosynthesis in different tissues, we compared DEGs between stem and rhizome, leaf and rhizome, and flower and rhizome, respectively (Figure 7 and Supplementary Table 6). We found that 67 genes involved in polysaccharide biosynthesis showed significant differences in expression, such as *MPI*, *GMPP*, *GMDS*, *GALE*, and *RHM* (Figure 7). Interestingly, the transcript levels of *GMPP* (*DN3245_c1_g2*), *GMDS* (*DN608_c0_g1*), and *RHM* (*DN155122_c0_g1*) were lower in stem, leaf, and flower compared to these of rhizome, respectively. Moreover, multiple genes involved in sucrose metabolism, including sucrose synthesis and degradation, were also discovered, such as *SPS*, *SUS*, and *INV*. As expected, the transcript levels of *SPS* (*DN19938_c0_g1*) and *SUS* (*DN15329_c0_g1*) were higher in the leaf than in other tissues. Similarly, most of the genes involved in the glycolysis/gluconeogenesis pathway, such as *ALDO*, *FBP*, *GPI*, *PGM*, and *UGP2* transcript levels, showed a similar tendency. Besides, *PGM* (*DN27690_c0_g1* and *DN5147_c0_g1*), *GPI* (*DN12212_c0_g1*), *FBP* (*DN17750_c0_g1*), and *UGP2* (*DN2305_c0_g1* and *DN2305_c0_g2*) were mainly expressed in the flower tissues of *P. cyrtonema* Hua.

### Identification of genes involved in sugar transport

**FIGURE 8:**
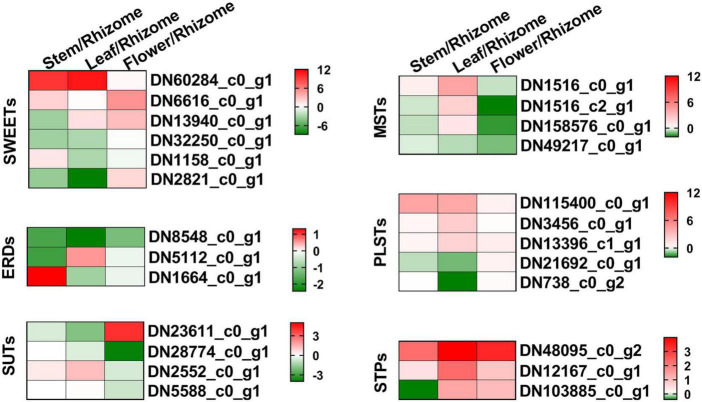
Heat map illustrating major expression profiles of differentially expressed genes (DEGs) related to the sugar transport. Sugar will eventually be exported transporters, SWEET; early response to dehydration, ERD; H + /sucrose transporters, SUT/SUC; monosaccharide transporters, MST; plastidic glucose transporter, PLST; sugar transport protein, STP. Red indicates high expression, white indicates intermediate expression, and green indicates low expression.

Sugar transporters play key roles in source-sink dynamics, which is important for carbon distribution and plant growth (Hennion et al., 2019). Thus, we analyzed the expression levels of *SWEETs*, *SUTs*, *ERDs*, *MSTs*, *PLSTs*, and *STPs* in the different tissues of *P. cyrtonema* Hua (Figure 8 and Supplementary Table 7). As shown in Figure 8, *SWEET* (*DN60284_c0_g1*) was mainly expressed in both leaf and stem, *SWEET* (*DN6616_c0_g1*) was mainly expressed in both stem and flower, and other *SWEETs* and *ERDs* were mainly expressed in stem and/or rhizome. *SUT* (*DN6616_c0_g1*) was mainly expressed in both rhizome and flower, and *SUT* (*DN2552_c0_g1*) was mainly expressed in both leaf and stem. Additionally, most of the monosaccharide transporters (*MSTs*), plastidic glucose transporters (*PLSTs*), and sugar transport proteins (*STPs*) were mainly expressed in the leaf. Combined with the content of sugar compositions, the above results indicated that these transporters might play important roles in the source-sink dynamics, especially in the accumulation of polysaccharides.

### Correlation between the polysaccharide accumulation and expression levels of genes related to polysaccharide biosynthesis and sugar transporters in different tissues

**FIGURE 9:**
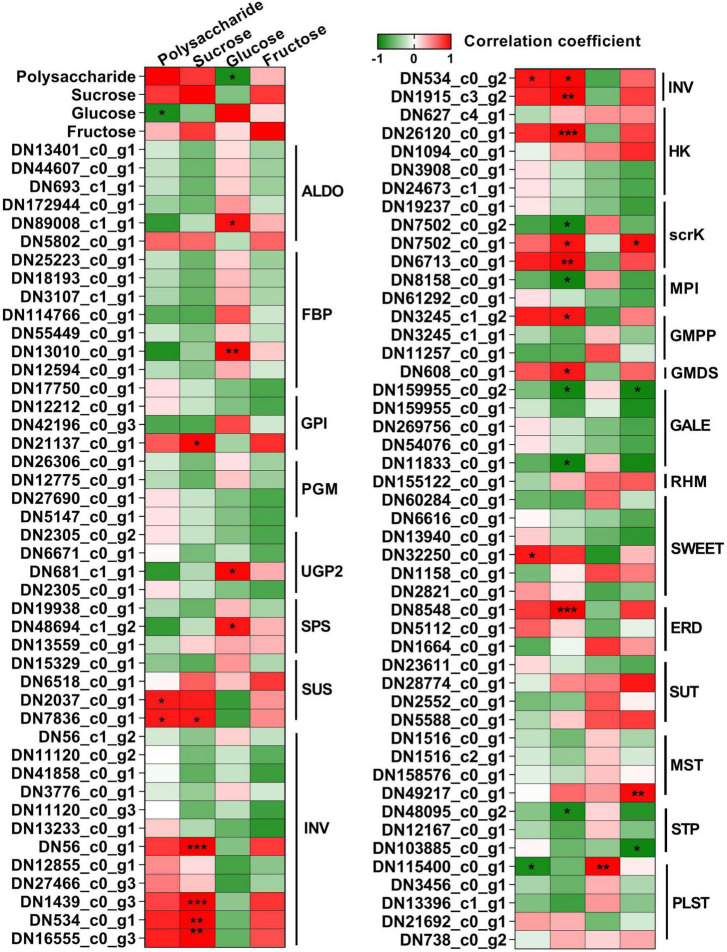
Heat map of the correlation coefficients between polysaccharide, sucrose, glucose, and fructose and the expression levels of genes related to sugar metabolism and transport in different tissues. Asterisks represent statistical significance determined by Student’s *t*-test (*P < 0.05, **P < 0.01, and ***P < 0.001).

To get further insight into the regulation of polysaccharide accumulation, we next analyzed the correlation between polysaccharide, sucrose, glucose, and fructose contents and the expression levels of genes related to polysaccharide biosynthesis and sugar transporters in the rhizome, stem, leaf, and flower tissues (Figure 9). Here, we found sucrose content was generally and positively correlated with polysaccharide content (*r* = 0.79), while glucose content showed a significant negative correlation (*r* = −0.90). There was a highly significant correlation between the expression levels of *GPI* (*DN21137_c0_g1*), *SUS* (*DN7836_c0_g1*), *INV* (*DN56_c0_g1*, *DN1439_c0_g3*, *DN534_c0_g1*, *DN16555_c0_g3*, *DN534_c0_g2*, and *DN1915_c3_g2*), *HK* (*DN26120_c0_g1*), *scrK* (*DN7502_c0_g2*, *DN7502_c0_g1*, and *DN6713_c0_g1*), *MPI* (*DN8158_c0_g1*), *GMPP* (*DN3245_c1_g2*), *GMDS* (*DN608_c0_g1*), *GALE* (*DN159955_c0_g2* and *DN11833_c0_g1*), *ERD* (*DN8548_c0_g1*), and sucrose content. We also observed that the expression of *ALDO* (*DN89008_c1_g1*), *FBP* (*DN13010_c0_g1*), *UGP2* (*DN681_c1_g1*), and *SPS* (*DN48694_c1_g2*) showed high positive correlation with the glucose content. Besides, the expression of *scrK* (*DN7502_c0_g1*) and *GALE* (*DN159955_c0_g2*) was positively and negatively correlated with fructose content, respectively. Moreover, the expression levels of *SUS* (*DN2037_c0_g1* and *DN7836_c0_g1*), *INV* (*DN534_c0_g2*), *SWEET* (*DN32250_c0_g1*), and *PLST* (*DN32250_c0_g1*) genes significantly correlated with polysaccharide content. These results suggested that the above genes were involved in the polysaccharide biosynthesis in *P. cyrtonema* Hua.

### Identification of transcription factors involved in polysaccharide accumulation

**FIGURE 10:**
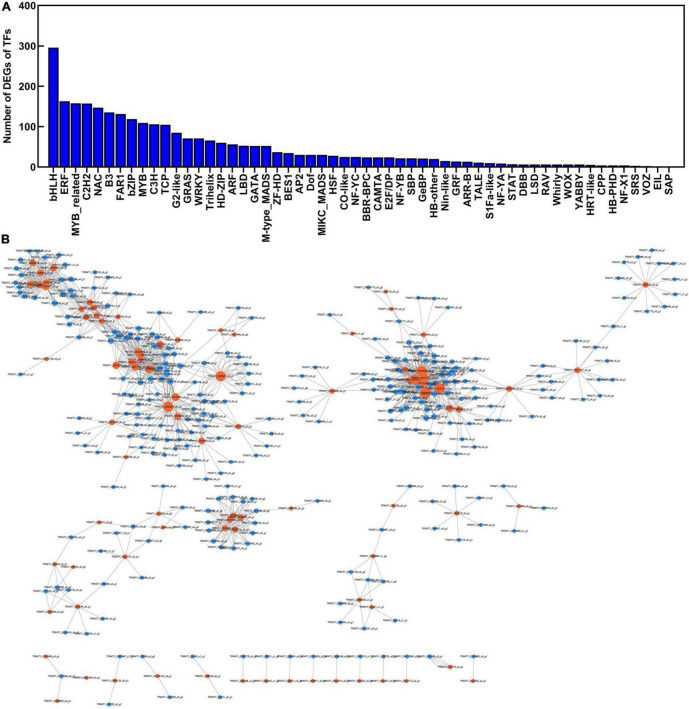
The co-expression network was constructed according to the differentially expressed transcription factors and sugar metabolism and transport genes in different tissues of *Polygonatum cyrtonema* Hua. **(A)** Type and number of transcription factors (TFs) of *P. cyrtonema* Hua. **(B)** The correlation coefficients of expression levels between transcription factors and sugar metabolism and transport in different tissues.

To explore the transcriptional regulation of genes related to polysaccharide accumulation, we next analyzed the transcription factors (TFs) in the rhizome, stem, leaf, and flower tissues of *P. cyrtonema* Hua. We found that 2,728 TFs were significantly up/downregulated (Figure 10). The major TF families were screened, including bHLH (296 unigenes), ERF (163 unigenes), MYB_related (158 unigenes), C2H2 (157 unigenes), NAC (147 unigenes), B3 (135 unigenes), FAR (131 unigenes), bZIP (119 unigenes), MYB (109 unigenes) groups (Figure 10A and Supplementary Table 8). Then, we constructed the co-expression network according to the differentially expressed transcription factors and sugar metabolism and transport genes (Figure 10B). There were 87 genes related to sugar metabolism and transport genes that presented very high correlations. For example, the *UGP2* (*DN681_c1_g1*) expression was highly and positively correlated with *bZIP* (*DN16926_c0_g1*), *bHLH* (*DN918_c1_g1*), and *ERF* (*DN25257_c0_g1*) expression (*r* = 0.96, 0.95, 0.98, *p* < 0.001). These results suggested that transcription factors might be involved in polysaccharide accumulation by regulating the expression of sugar metabolism and transport genes.

## Discussion

### Sugar accumulation pattern in different tissues of *Polygonatum cyrtonema* Hua

As bioactive ingredients, polysaccharides are of great value in the merits of medicinal plants (Li et al., 2021). Because rhizomes are the harvested targets, some studies have shown that rhizomes of *P. cyrtonema* Hua accumulated high content of polysaccharides, and unearthed several key genes involved in polysaccharide biosynthesis pathway from rhizomes at different ages by transcriptome sequencing (Li et al., 2022). Besides, polysaccharide accumulation is also highly cultivar- and tissue-dependent (Wang et al., 2019; Hu et al., 2022). For instance, Wang et al found that polysaccharide content was low in the leaf (1.62%) (Wang et al., 2019).

Most of the studies on polysaccharide accumulation were performed by examining the gene expression involved in the proposed pathway from sucrose to polysaccharide (Wang et al., 2019; Li et al., 2022). Furthermore, sucrose is the main form that is transported from source leaves to different sinks *via SWEETs*, *SUTs*, and/or *ERDs* transporters (Julius et al., 2017). Previous developments indicated that the role of sucrose synthase involved in sucrose import may play a role in directing carbon toward polysaccharide biosynthesis (Koch, 2004). Therefore, polysaccharide accumulation is dynamic and highly regulated. However, whether or how sucrose metabolism is involved in polysaccharide accumulation of shoots and rhizomes remains to be investigated in *P. cyrtonema* Hua.

In this report, we clearly observed that polysaccharides highly accumulated in rhizome tissue, which is consistent with the previously published results (Figure 3A; Wang et al., 2019). In addition, our results also indicated that sucrose and fructose levels were higher in the rhizome than in stem, leaf, and flower tissues (Figures 3B,D). In contrast, glucose content was lower in both the rhizome and flower of *P. cyrtonema* Hua (Figure 3C). Then, we analyzed the correlation between polysaccharide content and sucrose, glucose, or fructose in different tissues, respectively (Figure 9). These results demonstrated that the contents of sucrose and glucose were generally correlated with polysaccharide content. This was consistent with the regulation of sugar metabolism in rhizomes of *P. cyrtonema* Hua and *P. sibiricum* Red (Hu et al., 2022). Sugars were involved in most of the metabolic and signaling pathways controlling plant growth and development, as well as the responses to abiotic stresses. For example, leaf-derived sucrose accumulated in large amounts in the hypocotyls of tomato plants under flooding stress conditions (Mignolli et al., 2021). The above results suggested that sucrose metabolism might be involved in regulating the polysaccharide accumulation, which might play a critical role in tolerance to abiotic stresses in *P. cyrtonema* Hua.

### Response networks of the expression of polysaccharide biosynthesis and sugar transporter genes

The previous studies showed that a few hundred unigenes were involved in the sugar metabolism pathway. They found that the expression levels of unigenes encoding sacA, RHM, MPI, GMDS, TSTA3, UER1, and UGE enzymes were higher in rhizomes compared to leaves, while the expression levels of unigenes encoding *HK*, *scrK*, *UGP2*, *GPI*, *GMPP*, *GALE*, *UXE*, and *UGDH* were higher in leaves (Wang et al., 2019; Li et al., 2022). Combined with the sugar accumulation pattern in the above results, these findings led us to explore the regulation of the expression of polysaccharide biosynthesis genes in response to polysaccharide accumulation in rhizome, stem, leaf, and flower during the growth of *P. cyrtonema* Hua.

In this report, we analyzed the correlation between the expression of polysaccharide and sucrose biosynthesis pathway genes and polysaccharide, sucrose, or glucose content in rhizome, stem, leaf, and flower tissues, respectively. These results demonstrated that the expression of polysaccharide biosynthesis pathway genes is generally correlated with polysaccharide content in rhizomes (Figures 3, 7). Additionally, there were several unigenes, such as *HK* (*DN3908_c0_g1* and DN24673*_c1_g1*), *MPI* (*DN61292_c0_g1*), and *GALE* (*DN259756_c0_g1* and *DN54076_c0_g1*), specifically expressed in flowers, which are beneficial to polysaccharide biosynthesis in flowers (Figure 7). These results suggested that polysaccharide accumulation is generally regulated at the transcription level of polysaccharide biosynthesis genes.

In plants, sucrose, as an important form, is delivered to different tissues for metabolism and storage by the phloem (Hennion et al., 2019). This is apoplastically mediated through *SWEET*, *SUT/SUC*, and *EDR6* transporters (Stadler et al., 1995; Kiyosue et al., 1998; Milne et al., 2018; Chen et al., 2010, 2012). The molecular mechanisms of sugar transport, especially sucrose transport, are largely unknown in *P. cyrtonema* Hua. In this study, we found that there were several DEGs involved in sugar transport among rhizome, stem, leaf, and flower tissues (Figure 8). For example, *SWEET* (*DN60284_c0_g1*) was specifically expressed in both leaf and stem, while *SWEET* (*DN2821_c0_g1*) was specifically expressed in the rhizome. *SUT* (*DN23611_c0_g1*) was specifically expressed in the flower. These results suggested that the above genes showed different roles in sugar efflux and/or uptake, thereby participating in polysaccharide biosynthesis. Previous results showed that *OsSWEET13* and *OsSWEET15* genes modulated sucrose transport and distribution to maintain sugar homeostasis in response to drought and salinity stresses (Mathan et al., 2021b). It was also found that *AtSWEET17* was predicted to act as a vacuolar fructose importer, with a function in sequestering excess cytosolic fructose into vacuoles for storage in roots (Chardon et al., 2013). *AtSWEET16* overexpression increased the vacuolar sucrose and glucose accumulation to improve the freezing tolerance in *Arabidopsis* (Klemens et al., 2013). Furthermore, *AtSWEET15* was strongly induced by osmotic stresses, including cold, high salinity, and drought (Seo et al., 2011). It is also worth noting that both glucose content and plastidic glucose transporter (*PLST* and *DN115400_c0_g1*) expression showed a significantly negative correlation with polysaccharide content. Thus, the biological functions of these transporters should be further elucidated in *P. cyrtonema* Hua in the future.

### Putative key genes involved in the regulation of the sugar metabolism and homeostasis

In this study, we observed that the expression of sucrose synthesis and decomposition genes correlated differently with sucrose and polysaccharide contents among the different tissues (Figure 9). For example, *SUS* (*DN2037_c0_g1* and *DN7836_c0_g1*) and *INV* (*DN534_c0_g2*) transcripts positively correlated with the polysaccharide content. Emerging evidence had shed light on the potential role of *INVs* in the response to abiotic stress in various plant species (Dahro et al., 2016). Therefore, the vital role of *INV* in alleviating the abiotic stress in *P. cyrtonema* Hua needs to be explored in the future. Furthermore, the correlation between sucrose contents and the expression of polysaccharide biosynthesis genes was also discovered, such as *MPI* (*DN8158_c0_g1*), *GMPP* (*DN3245_c0_g2*), *GMDS* (*DN608_c0_g1*), and *GALE* (*DN159955_c0_g2* and *DN11833_c0_g1*) (Figure 9). Correlations between polysaccharide content and the expression of *MPI*, *GMPP*, *GMDS*, and *GALE* genes were observed by other reports in *P. cyrtonema* Hua (Wang et al., 2019; Li et al., 2022). Therefore, these results suggested that sucrose might be transported by *SWEET* (*DN32250_c0_g1*) to rhizomes, and then decomposed by *INV* to promote polysaccharide biosynthesis. Furthermore, several studies revealed that various transcription factors are involved in the regulation of sugar metabolism pathways which overcome the negative effects of abiotic stress (Kumar et al., 2022). For example, *MaRAP2-4*, an ERF transcription factor, activated *SWEET10* that directly or indirectly assists in sugar accumulation, thus enhancing tolerance to waterlogging, drought, and salinity (Phukan et al., 2018). Future research will focus on the functional role of these key genes involved in polysaccharide accumulation and their roles in responses to abiotic stress in *P. cyrtonema* Hua. Other studies suggested that gas signaling molecules and hormones also enhanced plant tolerance to abiotic stress by regulating sugar metabolism (Wei et al., 2015; Yang et al., 2016; Chen et al., 2020; Rangani et al., 2020; Gu et al., 2021, 2022; de Bont et al., 2022). For example, exogenous melatonin application improved the tolerance to salinity and drought stresses *via* modulating the starch/sucrose metabolism in soybean (Wei et al., 2015). Therefore, the roles of gas signaling molecules and hormones involved in polysaccharide accumulation are also worth in-depth study in *P. cyrtonema* Hua.

## Conclusion

**FIGURE 11:**
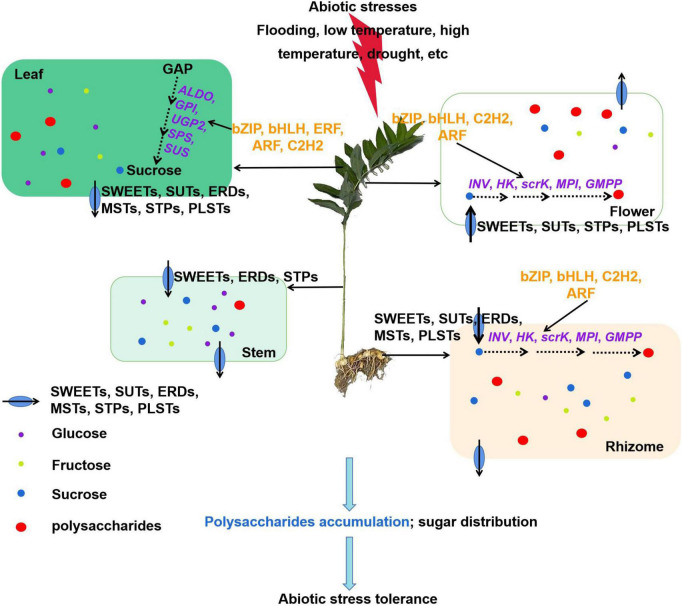
A model explaining the networks of transcriptional regulation of polysaccharide accumulation and their possible mechanisms in response to abiotic stress in *Polygonatum cyrtonema* Hua. Sucrose is mainly synthesized by the upregulation of *ALDO*, *GPI*, *UGP2*, *SPS*, and *SUS* genes in the leaf. Then, a high amount of sucrose and several monosaccharides is transported to the stem by *SWEET*s, *ERD*s, and/or *STP*s genes, and then get unloaded into the rhizome and flower tissues due to the functions of *SWEET*s, *SUTs*, *MST*s, *ERD*s, *PLST*s, and/or *STP*s genes. Afterward, sucrose is catalyzed by *INV*, and their products glucose and fructose are used to synthesize polysaccharides. Furthermore, these key genes are possibly regulated by transcription factors, such as *bZIP*, *bHLH*, *ERF*, *ARF*, and *C2H2*, which are involved in the regulatory processes of abiotic stresses. In general, our study contributes to a comprehensive understanding of the transcriptional regulation of polysaccharide accumulation.

In this study, we explored a detailed regulatory network for polysaccharide accumulation in *P. cyrtonema* Hua. Our results showed that polysaccharides generally accumulated in rhizome, stem, leaf, and flower tissues. Moreover, the polysaccharide biosynthesis in rhizome and flower is closely related to sucrose metabolism and transport in the leaf and stem (Figure 11). Sucrose is mainly synthesized by the upregulation of *ALDO*, *GPI*, *UGP2*, *SPS*, and *SUS* genes in the leaf. Then, a high amount of sucrose and several monosaccharides are transported to the stem, and then get unloaded into the rhizome and flower tissues due to the functions of *SWEET*s, SUTs, *MST*s, *ERD*s, *PLST*s, and/or *STP* genes. Afterward, sucrose is catalyzed by *INV*, and their products glucose and fructose are used to synthesize polysaccharides. Furthermore, these key genes are possibly regulated by transcription factors, such as *bZIP*, *bHLH*, *ERF*, *ARF*, and *C2H2*, which are involved in the regulatory processes of abiotic stresses. To our knowledge, this is the first report to link sucrose metabolism and transport with polysaccharide accumulation in *P. cyrtonema* Hua. This study provided new insights into the molecular mechanism of polysaccharide biosynthesis and valuable genes involved in abiotic stress tolerance in *P. cyrtonema* Hua. Our results also suggested that as polysaccharides have various health effects, not only rhizome, but leaves and flowers can also be used to process the foods to meet the market demand.

## Data availability statement

Publicly available datasets were analyzed in this study. RNA-seq data of the tissues can be found in the publicly accessible NCBI under BioProject PRJNA878799.

## Author contributions

XD, ZC, and LC conceived the study and designed the experiments. LC, SX, YL, YZ, FZ, LD, JC, KW, and SC performed the experiments. LL and YW participated in the preparation of plant materials. LC wrote the manuscript. XD and ZC revised and finalized the manuscript. All authors read and approved the final version of the manuscript.

## Footnotes

1
http://www.genome.jp/kegg

2
http://geneontology.org
