# Supplementary material for: Identification of key gene networks controlling polysaccharide accumulation in different tissues of Polygonatum cyrtonema Hua by integrating metabolic phenotypes and gene expression profiles
Source: Front Plant Sci. 2022 Sep 29;13:1012231. doi: 10.3389/fpls.2022.1012231 (PMC9558278; doi:10.3389/fpls.2022.1012231)
Supplement: Supplementary file 1 [file Data_Sheet_1.docx]

**Supplementary Materials**

**Table S1** Summary of sequencing quality.

| Sample name | Raw reads | Clean reads | Clean bases | Q20% | Q30% |
| --- | --- | --- | --- | --- | --- |
| Rhizome1 | 44788178 | 42336126 | 6350418900 | 97.45 | 92.94 |
| Rhizome2 | 53245850 | 50330176 | 7549526400 | 97.6 | 93.34 |
| Rhizome3 | 48520318 | 45824008 | 6873601200 | 97.24 | 92.51 |
| Stem1 | 51728176 | 48455736 | 7268360400 | 97.53 | 93.1 |
| Stem2 | 54180160 | 50732110 | 7609816500 | 97.28 | 92.57 |
| Stem3 | 50775870 | 47565150 | 7134772500 | 97.24 | 92.42 |
| Leaf1 | 48735228 | 45718700 | 6857805000 | 97.56 | 93.19 |
| Leaf2 | 48049580 | 45146228 | 6771934200 | 97.44 | 92.88 |
| Leaf3 | 51562406 | 48334336 | 7250150400 | 97.7 | 93.58 |
| Flower1 | 55048918 | 52068538 | 7810280700 | 97.75 | 93.7 |
| Flower2 | 48085880 | 45415296 | 6812294400 | 97.67 | 93.43 |
| Flower3 | 48299552 | 45579596 | 6836939400 | 97.59 | 93.26 |

**Table S2** Length distribution of assembled transcripts and unigenes

| Nucleotide length | Transcript | Unigene |
| --- | --- | --- |
| Total Length (bp) | 401227759 | 157174631 |
| Sequence Number | 393729 | 182700 |
| Max. Length (bp) | 15842 | 15842 |
| Mean Length (bp) | 1019.05 | 860.29 |
| N50 (bp) | 1424 | 1142 |
| N50 Sequence No. | 84566 | 38618 |
| N90 (bp) | 453 | 395 |
| N90 Sequence No. | 285176 | 136992 |
| GC% | 45.15 | 44.78 |

**Table S3** Summary of the unigenes annotated in six public databases

| Database | Number | Annotated unigene ratio(%) |
| --- | --- | --- |
| NR | 90381 | 49.47 |
| GO | 39774 | 21.77 |
| KEGG | 37737 | 20.66 |
| Pfam | 55775 | 30.53 |
| eggNOG | 74522 | 40.79 |
| Swissprot | 68086 | 37.27 |

**Table S4** The 11 pathway classifications for the carbohydrate metabolism of DEGs by KEGG analysis

| Pathway | DEGs Stem/Rhizome | DEGs  Leaf/Rhizome | DEGs  Flower/Rhizome |
| --- | --- | --- | --- |
| Glycolysis/Gluconeogenesis | 44 | 100 | 190 |
| Starch and sucrose metabolism | 52 | 103 | 206 |
| Glyoxylate and dicarboxylate metabolism | 32 | 85 | 181 |
| Amino sugar and nucleotide sugar metabolism | 32 | 66 | 166 |
| Galactose metabolism | 31 | 57 | 140 |
| Fructose and mannose metabolism | 27 | 48 | 116 |
| Pentose phosphate pathway | 26 | 56 | 94 |
| Pentose and glucuronate interconversions | 18 | 32 | 114 |
| Inositol phosphate metabolism | 14 | 23 | 72 |
| Ascorbate and aldarate metabolism | 13 | 28 | 64 |
| Butanoate metabolism | 6 | 17 | 68 |

**Table S5** The 12 pathway classifications for the polysaccharide metabolism by GO analysis

| Pathway | DEGs Stem/Rhizome | DEGs  Leaf/Rhizome | DEGs  Flower/Rhizome |
| --- | --- | --- | --- |
| polysaccharide metabolic process | 111 | 205 | 222 |
| polysaccharide biosynthetic process | 52 | 100 | 61 |
| polysaccharide catabolic process | 41 | 80 | 133 |
| cellular polysaccharide metabolic process | 81 | 153 | 164 |
| cellular polysaccharide catabolic process | 12 | 35 | 40 |
| cellular polysaccharide biosynthetic process | 48 | 91 | 59 |
| cell wall polysaccharide metabolic process | 23 | 39 | 81 |
| cell wall polysaccharide catabolic process | 4 | 8 | 48 |
| cell wall polysaccharide biosynthetic process | 9 | 19 | 9 |
| extracellular polysaccharide metabolic process | 0 | 1 | 7 |
| carbon-oxygen lyase activity, acting on polysaccharides | 5 | 6 | 14 |
| polysaccharide binding | 17 | 36 | 19 |

**Table S6** Expression of unigenes related to the polysaccharide biosynthesis

| Gene ID | Stem/Rhizome | Leaf/Rhizome | Flower/Rhizome |
| --- | --- | --- | --- |
| DN13401_c0_g1 | 3.95240398 | 11.15910918 | -1.300915439 |
| DN44607_c0_g1 | 5.922494041 | 10.13075228 | 4.822988024 |
| DN693_c1_g1 | 6.503782244 | 10.52500441 | 5.494696498 |
| DN172944_c0_g1 | 2.845269699 | 5.079909636 | -0.393048591 |
| DN89008_c1_g1 | 2.555218002 | 0.82211355 | -0.037245218 |
| DN5802_c0_g1 | -1.928605538 | -0.388789145 | -2.681819929 |
| DN25223_c0_g1 | 5.01399746 | 9.176410697 | 4.311229005 |
| DN18193_c0_g1 | 4.684035422 | 7.931402252 | -0.519302693 |
| DN3107_c1_g1 | 2.870288156 | 5.491811923 | 0.276626258 |
| DN114766_c0_g1 | 1.921827081 | 2.840629107 | 0.257271718 |
| DN55449_c0_g1 | -0.139234368 | 2.818404651 | -2.857829447 |
| DN13010_c0_g1 | 3.126635702 | 1.5669698 | -0.58290811 |
| DN12594_c0_g1 | 0.038782412 | 1.615469535 | -0.304339348 |
| DN17750_c0_g1 | 1.554728119 | 0.034179459 | 11.38385848 |
| DN12212_c0_g1 | 4.770806097 | 2.866895508 | 12.42078553 |
| DN42196_c0_g3 | 0.936205999 | 1.740743613 | 0.32284989 |
| DN21137_c0_g1 | -1.052844892 | -1.520902056 | -0.863438062 |
| DN26306_c0_g1 | 1.296854558 | 8.436123248 | 0.296916545 |
| DN12775_c0_g1 | 1.065877209 | 3.861961022 | 0.666535919 |
| DN27690_c0_g1 | 0.55262439 | 0.165640184 | 7.667451979 |
| DN5147_c0_g1 | 3.808910065 | 0.033118257 | 10.2507745 |
| DN2305_c0_g2 | 4.364230325 | 1.708099661 | 10.86420506 |
| DN6671_c0_g1 | -0.646198229 | 1.560004176 | 0.80843294 |
| DN681_c1_g1 | 1.416582462 | 0.799497921 | -0.249011683 |
| DN2305_c0_g1 | 3.82217385 | 0.669722128 | 10.57101303 |
| DN19938_c0_g1 | 2.745379242 | 5.725718077 | 0.377178271 |
| DN48694_c1_g2 | 5.874896823 | 2.403879322 | 0.624375788 |
| DN13559_c0_g1 | 0.433170884 | -2.546275214 | 0.547523275 |
| DN15329_c0_g1 | 4.999600918 | 7.111222596 | 2.114231304 |
| DN6518_c0_g1 | -0.732291687 | -1.467173231 | -0.116857499 |
| DN2037_c0_g1 | -2.497781259 | -2.812963219 | -0.501197171 |
| DN7836_c0_g1 | -2.556007666 | -3.41793302 | -0.634891397 |
| DN56_c1_g2 | -0.222696139 | 2.254551536 | -1.31950758 |
| DN11120_c0_g2 | -0.876196993 | 2.20757852 | 1.161110129 |
| DN41858_c0_g1 | -0.47089491 | 2.025870338 | 1.400775795 |
| DN3776_c0_g1 | -0.332402494 | 1.966074476 | -1.634128651 |
| DN11120_c0_g3 | -0.765280299 | 1.763258926 | 1.27469971 |
| DN13233_c0_g1 | -1.511557424 | 1.148779047 | 2.329191423 |
| DN56_c0_g1 | -1.981248128 | -3.67874745 | -1.475165576 |
| DN12855_c0_g1 | -4.428297747 | -4.21112946 | 1.634841598 |
| DN27466_c0_g3 | -4.078579453 | -4.307215161 | 1.100947255 |
| DN1439_c0_g3 | -1.839738218 | -4.311598578 | -1.496981383 |
| DN534_c0_g1 | -4.799803555 | -5.168563072 | -2.712002185 |
| DN16555_c0_g3 | -3.908771552 | -5.34697815 | -1.942469544 |
| DN534_c0_g2 | -4.936062422 | -6.211897016 | -1.407276111 |
| DN1915_c3_g2 | -4.914573375 | -6.876956774 | -3.24281327 |
| DN627_c4_g1 | 0.445016914 | -2.041891377 | 0.126691741 |
| DN26120_c0_g1 | -1.180469364 | -1.052666992 | -0.627534645 |
| DN1094_c0_g1 | -0.269454734 | -0.031356343 | -1.010268409 |
| DN3908_c0_g1 | 2.449029144 | -Inf | 10.13144544 |
| DN24673_c1_g1 | 1.67542913 | -Inf | 7.49770626 |
| DN19237_c0_g1 | 1.190998589 | 3.809720182 | 6.941631926 |
| DN7502_c0_g2 | 1.036271685 | 1.932321647 | 1.224326402 |
| DN7502_c0_g1 | -1.498099523 | -1.922015732 | -3.334953108 |
| DN6713_c0_g1 | -3.217632671 | -3.106662437 | -2.192204337 |
| DN8158_c0_g1 | 0.884313704 | 1.930840445 | 1.387648999 |
| DN61292_c0_g1 | -0.316174299 | 0.122916712 | 9.708349342 |
| DN3245_c1_g2 | -1.481196676 | -1.356888674 | -0.362694514 |
| DN3245_c1_g1 | 0.213346964 | 1.937907461 | 0.515363352 |
| DN11257_c0_g1 | 2.027137246 | 2.906573695 | 0.339962524 |
| DN608_c0_g1 | -0.837524527 | -1.097690935 | -0.19968951 |
| DN159955_c0_g2 | 2.699162519 | 4.164771482 | 3.706715104 |
| DN159955_c0_g1 | 1.279826981 | 4.10730775 | 3.67064783 |
| DN269756_c0_g1 | 1.700795268 | 2.128090065 | 9.600694032 |
| DN54076_c0_g1 | 3.023756005 | 1.862031725 | 8.674110292 |
| DN11833_c0_g1 | 1.080234349 | 1.797674039 | 1.854488746 |
| DN155122_c0_g1 | 0.457618269 | -1.718719834 | -0.575110648 |

**Table S7** Expression of unigenes related to sugar transport

| Gene ID | Stem/Rhizome | Leaf/Rhizome | Flower/Rhizome |
| --- | --- | --- | --- |
| DN60284_c0_g1 | 9.496960122 | 10.92323347 | 0.296916545 |
| DN6616_c0_g1 | 2.032974809 | 0.097747983 | 5.04706223 |
| DN13940_c0_g1 | -3.351819289 | 1.443090249 | 3.017140545 |
| DN32250_c0_g1 | -3.302020051 | -2.822840603 | -0.157708641 |
| DN1158_c0_g1 | 1.22338254 | -2.821964007 | -0.483664706 |
| DN2821_c0_g1 | -3.691755109 | -8.863255965 | 1.815328169 |
| DN8548_c0_g1 | -1.731301441 | -2.433523814 | -1.277312162 |
| DN5112_c0_g1 | -1.832839953 | 0.530810547 | -0.172730694 |
| DN1664_c0_g1 | 1.309348158 | -0.901108275 | -0.193069215 |
| DN23611_c0_g1 | -0.617952936 | -1.946761627 | 4.057360988 |
| DN28774_c0_g1 | -0.01936457 | -0.555397773 | -3.920364356 |
| DN2552_c0_g1 | 0.425412818 | 1.24269401 | -0.64774903 |
| DN5588_c0_g1 | -0.029954375 | 0.049042988 | -0.826764844 |
| DN1516_c0_g1 | 0.79786392 | 4.236827686 | -0.459068661 |
| DN1516_c2_g1 | -0.391854562 | 2.147779401 | -1.998604874 |
| DN158576_c0_g1 | -0.507286759 | 1.202126308 | -1.599768755 |
| DN49217_c0_g1 | -0.275430096 | -0.568873387 | -1.03921222 |
| DN48095_c0_g2 | 2.264271564 | 3.978166293 | 3.36780567 |
| DN12167_c0_g1 | 0.481971497 | 2.308490536 | 0.892468661 |
| DN103885_c0_g1 | -0.402165813 | 1.37855404 | 1.05228182 |
| DN115400_c0_g1 | 4.197952309 | 4.001675849 | 0.6264592 |
| DN3456_c0_g1 | 0.458300232 | 2.307177016 | 0.096296811 |
| DN13396_c1_g1 | 0.459642843 | 2.056952797 | 0.806896136 |
| DN21692_c0_g1 | -0.520212952 | -1.070607182 | 0.600709016 |
| DN738_c0_g2 | 0.002932137 | -1.955282585 | 0.219946511 |

**Table S8** Expression of unigenes related to TFs

| Gene ID | Stem/Rhizome | Leaf/Rhizome | Flower/Rhizome |
| --- | --- | --- | --- |
| DN60284_c0_g1 | 9.496960122 | 10.92323347 | 0.296916545 |
| DN6616_c0_g1 | 2.032974809 | 0.097747983 | 5.04706223 |
| DN13940_c0_g1 | -3.351819289 | 1.443090249 | 3.017140545 |
| DN32250_c0_g1 | -3.302020051 | -2.822840603 | -0.157708641 |
| DN1158_c0_g1 | 1.22338254 | -2.821964007 | -0.483664706 |
| DN2821_c0_g1 | -3.691755109 | -8.863255965 | 1.815328169 |
| DN8548_c0_g1 | -1.731301441 | -2.433523814 | -1.277312162 |
| DN5112_c0_g1 | -1.832839953 | 0.530810547 | -0.172730694 |
| DN1664_c0_g1 | 1.309348158 | -0.901108275 | -0.193069215 |
| DN23611_c0_g1 | -0.617952936 | -1.946761627 | 4.057360988 |
| DN28774_c0_g1 | -0.01936457 | -0.555397773 | -3.920364356 |
| DN2552_c0_g1 | 0.425412818 | 1.24269401 | -0.64774903 |
| DN5588_c0_g1 | -0.029954375 | 0.049042988 | -0.826764844 |
| DN1516_c0_g1 | 0.79786392 | 4.236827686 | -0.459068661 |
| DN1516_c2_g1 | -0.391854562 | 2.147779401 | -1.998604874 |
| DN158576_c0_g1 | -0.507286759 | 1.202126308 | -1.599768755 |
| DN49217_c0_g1 | -0.275430096 | -0.568873387 | -1.03921222 |
| DN48095_c0_g2 | 2.264271564 | 3.978166293 | 3.36780567 |
| DN12167_c0_g1 | 0.481971497 | 2.308490536 | 0.892468661 |
| DN103885_c0_g1 | -0.402165813 | 1.37855404 | 1.05228182 |
| DN115400_c0_g1 | 4.197952309 | 4.001675849 | 0.6264592 |
| DN3456_c0_g1 | 0.458300232 | 2.307177016 | 0.096296811 |
| DN13396_c1_g1 | 0.459642843 | 2.056952797 | 0.806896136 |
| DN21692_c0_g1 | -0.520212952 | -1.070607182 | 0.600709016 |
| DN738_c0_g2 | 0.002932137 | -1.955282585 | 0.219946511 |
